# Effectiveness of Physical Activity with Sports Scientist (PASS) Programme Among Patients with Non-Communicable Diseases in Primary Care: A Randomised Controlled Trial

**DOI:** 10.3390/medsci13040279

**Published:** 2025-11-21

**Authors:** Apichai Wattanapisit, Poramet Hemarachatanon, Kamlai Somrak, Saranrat Manunyanon, Sanhapan Wattanapisit, Phiphat Khlongdi, Kiattisak Pechpan, Areekul Amornsriwatanakul, Piyawat Katewongsa, Sorawat Sangkaew, Polathep Vichitkunakorn, Ping Yein Lee, Siti Nurkamilla Ramdzan, Hani Salim, Chirk Jenn Ng, Mark Stoutenberg

**Affiliations:** 1 Department of Clinical Medicine, School of Medicine, Walailak University, Nakhon Si Thammarat 80160, Thailand; 2 Family Medicine Clinic, Walailak University Hospital, Nakhon Si Thammarat 80160, Thailand; 3 The Excellent Center of Community Health Promotion, Walailak University, Nakhon Si Thammarat 80160, Thailand; 4 Department of Sport and Exercise Science, School of Medicine, Walailak University, Nakhon Si Thammarat 80160, Thailand; 5 Department of Community Nursing, School of Nursing, Walailak University, Nakhon Si Thammarat 80160, Thailand; 6 Department of Health Promotion, Walailak University Hospital, Nakhon Si Thammarat 80160, Thailand; 7 Center for Cultural and Sports Promotion, Walailak University, Nakhon Si Thammarat 80160, Thailand; 8 Family Medicine Unit, Thasala Hospital, Nakhon Si Thammarat 80160, Thailand; 9 College of Sports Science and Technology, Mahidol University, Nakhon Pathom 73170, Thailand; 10 Institute for Population and Social Research, Mahidol University, Nakhon Pathom 73170, Thailand; 11 Department of Social Medicine, Hatyai Hospital, Songkhla 90110, Thailand; 12 Department of Family and Preventive Medicine, Faculty of Medicine, Prince of Songkla University, Songkhla 90110, Thailand; 13 Health Policy Research Center (HPRC), Faculty of Medicine, Prince of Songkla University, Songkhla 90110, Thailand; 14 UMeHealth Unit, Faculty of Medicine, Universiti Malaya, Kuala Lumpur 50603, Malaysia; 15 Department of Primary Care Medicine, Faculty of Medicine, Universiti Malaya, Kuala Lumpur 50603, Malaysia; 16 Department of Family Medicine, Faculty of Medicine and Health Sciences, Universiti Putra Malaysia, Selangor 43400, Malaysia; 17 SingHealth Polyclinics, Singapore Health Services (SingHealth), Singapore 150167, Singapore; 18 SingHealth Duke-NUS Family Medicine Academic Clinical Programme, Duke-NUS Medical School, Singapore 169857, Singapore; 19 Department of Sport and Exercise Sciences, Durham University, Durham DH1 3LA, UK

**Keywords:** non-communicable disease, physical activity, primary care

## Abstract

**Objectives:** This study examined the effectiveness of a physical activity (PA) promotion intervention administered by a sports scientist as part of team-based care in a primary care setting. **Methods:** A randomised controlled trial was conducted. Physically inactive participants aged 35–70 years with non-communicable diseases (NCDs) were recruited. All participants received PA screening by a nurse and brief PA counselling by a physician. The intervention group also received a tailored PA programme at the first visit and monthly phone calls for 6–8 months (from visit 1 to visit 3). Outcome assessments by a sports scientist were performed for both groups at every visit (visit 1: baseline, visit 2: follow-up, visit 3: end-point, visit 4: continuing). Outcomes included meeting PA recommendations and weekly time spent in aerobic PA. An intention-to-treat analysis was applied. **Results:** Sixty participants were randomly allocated to each group. At visit 2 (months 3–4), significantly higher proportion of participants in the intervention group were meeting PA recommendations compared with the control group: aerobic PA (23.3% vs. 6.7%, *p* < 0.05), muscle-strengthening activity (31.7% vs. 0%, *p* < 0.001), and multicomponent PA (20.0% vs. 0%, *p* < 0.001). Median time spent in moderate- to vigorous-intensity PA (MVPA) was also higher (90 min/week vs. 60 min/week, *p* < 0.05). Weekly MVPA time increased significantly from baseline in both groups. **Conclusions:** Integrating a sports scientist into team-based care effectively improved short-term PA levels when intervention intensity was highest. The team-based care integrating sports scientists into primary care may enhance PA promotion for patients with NCDs.

## 1. Introduction

Physical activity (PA) interventions in primary care are effective at increasing moderate- to vigorous-intensity PA (MVPA) and improving the likelihood of achieving recommended PA levels [1]. However, only a small proportion of patients in primary care settings (37.9%) receive PA counselling from their primary care providers [2]. In comparison, patients with non-communicable diseases (NCDs) report higher rates of PA counselling: 65.5% for those with diabetes mellitus (DM), 56.8% for those with overweight or obesity, and 41.6% for those with hypertension (HT) [2]. Barriers to PA promotion in primary care practice include factors related to providers (e.g., knowledge and skills), patients (e.g., negative perceptions), and healthcare systems (e.g., limited consultation time) [3,4,5].

PA promotion in healthcare settings is an umbrella term that includes a number of methods (e.g., PA counselling by clinicians, PA counselling by support staff, co-location of exercise professionals, internal referrals, external referrals, environmental changes) [6,7,8]. A combination of approaches is recommended for promoting PA in primary care [9]. Brief interventions provided by healthcare providers constitute one approach, typically taking only a few minutes and including discussion, support, and follow-up [9]. PA referral schemes represent another approach, whereby healthcare providers formally refer patients to exercise professionals [9]. PA referral schemes with connectors are a further approach, in which primary care providers refer their patients to ‘connectors’ who serve as a bridge between primary care and the PA sector [9,10,11]. Co-location is an alternative approach that combines clinics with other health-related services to address referral limitations, improve patient adherence, and reduce the likelihood of dropouts [7].

Most PA promotion interventions delivered in primary care have been initiated in high-income countries [1,9]. In low- and middle-income countries, such as Thailand, the infrastructure for PA promotion interventions is not well established. PA referral schemes are uncommon, and there is a lack of certified exercise professionals trained to work with clinical populations [12]. These challenges raise an important question regarding an intervention to promote PA in clinical settings in Thailand. Moreover, characteristics of primary care vary across settings and contexts. Hospital-based ambulatory clinics are common in Thailand’s public hospitals. These hospital-based ambulatory clinics function as primary care settings along with other healthcare settings (e.g., community health centres (called sub-district health promoting hospitals) and private clinics) across the country. Considering the characteristics of hospital-based primary care, incorporating a sports scientist into the primary care team may enable the optimisation of a co-location model within a single clinic. This study aims to examine the effectiveness of a co-located sports scientist into an integrated care team in a hospital-based primary care setting.

## 2. Methods

A co-located sports scientist PA promotion intervention called Physical Activity with Sports Scientist (PASS) programme was implemented. The PASS study protocol was previously published [12]. This study followed the Consolidated Standards of Reporting Trials (CONSORT) [13]. The trial was registered with the Thai Clinical Trials Registry (TCTR20240314001) on 14 March 2024.

### 2.1. Patient and Public Involvement

The study protocol was consulted with a group of patient and public involvement consisting of six individuals who received NCD care at the study site. The study protocol was amended based on their comments before submission to the human research ethics committee.

### 2.2. Equity, Diversity, and Inclusion

This study was conducted in Southern Thailand, a region within a middle-income country. Participants of all biological sexes were included, with no exclusion based on gender identity, skin colour, socioeconomic status, culture, religion, or ethnicity. The author team reflects diversity in gender identity, cultural and religious backgrounds, and includes members from both low- and middle-income and high-income countries.

### 2.3. Study Design and Setting

A pragmatic randomised controlled trial (RCT) with 1:1 allocation ratio and two parallel groups was conducted at the Family Medicine Clinic, Walailak University, Thailand. The clinic is located within a hospital building (hospital-based clinic) and consists of a family physician and nursing staff. Access to laboratory tests and imaging services is available through the hospital facility. The clinic provides ambulatory care and is designed to deliver continuity of care, ensuring that patients with chronic conditions are seen by the same family physician. The family medicine team works closely with the home care team and three community health centres located in the surrounding sub-districts, which form the hospital’s catchment area. The study was conducted between March 2024 and May 2025.

### 2.4. Participants and Recruitment

Before a medical consultation, a nurse completed a short checklist form to identify eligible participants. Participants were physically inactive patients in regular clinic practice aged 35–70 years with at least one NCD, including type 2 DM (T2DM), HT, and dyslipidaemia (DLP). Physical inactivity was defined as <150 min/week of moderate-intensity aerobic PA or <75 min/week of vigorous intensity of aerobic PA [14]. Participants were excluded if they had uncontrolled NCDs (e.g., fasting plasma glucose ≥300 mg/dL, blood pressure ≥180/105 mmHg, muscle pain from lipid-lowering medication); signs and symptoms of cardiovascular diseases (e.g., chest pain, palpitation, ankle oedema); pregnancy or breast feeding; uncontrolled respiratory diseases (e.g., asthma, chronic obstructive pulmonary disease); drug interactions; movement limitations; or another study participant in the same household.

Subsequently, a research assistant met eligible patients in the waiting room, provided information about the PASS programme, and invited eligible patients to participate in the study. If the patient agreed to participate in the study, written informed consent was requested.

A total of 134 eligible patients were invited to participate in the study. Fourteen refused due to lack of interest (*n* = 10), lack of time (*n* = 3), and health concerns (i.e., knee pain) (*n* = 1).

### 2.5. Sample Size

A sample size calculator software, n4Studies (version 1.4.0), was used to calculate the sample size using binary data [15]. The proportion of patients meeting PA recommendation after interventions was estimated at 78% and 51% in the intervention and control groups, respectively [1]. Using a 1:1 allocation ratio, a type I error rate of 0.05, and a type II error rate of 0.2, a sample size of 49 was calculated for each group. Accounting for an estimated 20% dropout rate, a final sample size of 60 was established for each group.

### 2.6. Randomisation

Blocks of four allocation sequences were generated using a randomisation generator, Sealed Envelope (https://www.sealedenvelope.com/simple-randomiser/v1/lists) (accessed on 1 February 2024). by a research assistant. The physician (principal investigator: A.W.) was blinded to the block size and allocation sequence. The sports scientist was unblinded to the group allocation after the initial outcome assessment at visit 1. The data analysts were blinded to group allocation.

### 2.7. Interventions

**Figure 1:**
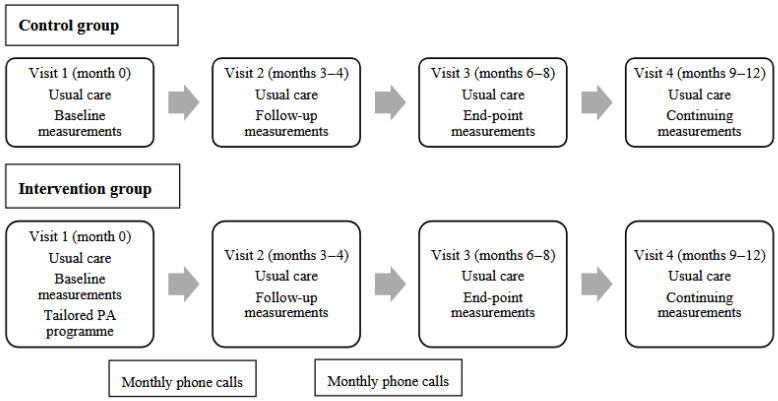
Overview and schedule of intervention activities.

All participants received PA screening by a nurse and brief PA counselling by a physician. At visit 1, participants in the intervention group received a tailored PA programme designed by a sports scientist based on the WHO recommendations separating PA components into FITT Pro (frequency, intensity, time, type, and progression) [14,16]. The PA programme included aerobic, muscle-strengthening and multicomponent PA. Details for flexibly completing the PA programme (e.g., 2–4 sets and 8–15 repetitions of muscle strengthening) were discussed based on participants’ ability and appropriateness. Short videos were played to illustrate recommended activities. The sports scientist also provided the participant with exercise equipment (e.g., a resistance band). Between visits 1 and 2 and visits 2 and 3, a sports scientist contacted participants via monthly phone calls (once a month) to ask about their PA participation and barriers experienced, to review the assigned programme, and to monitor abnormal symptoms or injuries (Figure 1).

### 2.8. Outcomes

The primary outcomes were meeting PA recommendations for: (i) aerobic activity (≥150 min/week of MVPA), (ii) muscle strengthening (≥2 times/week), and (iii) multicomponent activity (≥3 times/week) [14]. Secondary outcomes included (i) body composition (i.e., body weight, body mass index, fat mass, percentage of fat, muscle mass, and percentage of muscle mass) and (ii) health-related physical fitness (i.e., flexibility, muscle strength and endurance, and cardiovascular endurance).

### 2.9. Data Collection

The Thai translation of the Exercise Vital Sign (EVS) consisting of two questions: ‘on average, how many days per week do you engage in moderate to strenuous PA or exercise?’ and ‘on average, how many minutes per day do you engage in moderate to strenuous PA or exercise?’ was used to identify meeting PA recommendations at the nurse station before medical consultation [17,18].

During the medical consultation, the physician (A.W.) recorded the participants’ age (in years), sex (female or male), and NCDs (T2DM, HT, DLP: Yes or No). Checklists for brief PA counselling (type and intensity), medical screening, and stage of readiness to change were completed.

After the medical consultation, participants met a clinic nurse for their post-consultation summary and an appointment in the next 3–4 months, as per usual practice. Participants then walked to another room in the clinic area to meet the sports scientist.

### 2.10. Study Assessments

A series of assessments for both groups were conducted by the sports scientist at four visits after medical consultations. The sports scientist reviewed the EVS and classified participants as yes (≥150 min/week of MVPA) or no (<150 min/week of MVPA). Moderate or greater intensity of muscle-strengthening activity (yes: ≥2 times/week) and moderate or greater intensity of multicomponent PA (yes: ≥3 times/week) were asked verbally and recorded in the form. Body composition was measured using bioelectrical impedance analysis (Tanita model SC330P; Tanita, Tokyo, Japan). Physical fitness tests included flexibility, muscle strength and endurance, and cardiovascular endurance based on participant age. Based on the physical fitness tests, participants were categorised as pass (moderate, good, or very good) or fail (very low or low) according to standards for age and sex of the manual of Thailand Department of Physical Education [19].

### 2.11. Data Analysis

Categorical variables were presented as frequencies and percentages. Continuous variables were tested by the Shapiro–Wilk test for normality. Normally distributed variables were presented by means and standard deviations. Medians and interquartile ranges were used for non-normally distributed variables.

An intention-to-treat analysis was conducted. Measurements of missing participants and data were replaced using the most recent measurements. Analytic statistics were performed to analyse differences in baseline characteristics of participants and outcomes between the control and intervention groups. Categorical variables were analysed using Chi-square or Fisher’s exact tests. Continuous variables were analysed using the independent *t*-test or the Mann–Whitney U test for non-parametric statistics. Effect sizes and 95% confidence intervals (95%CI) for primary outcomes and any significant secondary outcomes were calculated using risk ratios (RR) or risk differences (RD) when the denominator was zero for categorical variables; Cohen’s d for normally distributed continuous variables; and Cliff’s delta for non-normally distributed continuous variables. A two-way analysis of variance (ANOVA) was conducted to examine longitudinal outcome changes between the visits and the two groups. Post hoc comparisons were performed using the Tukey multiple comparison test to explore differences in outcomes within each group across different visits. A statistic software, R V.4.0.2 (RStudio, Boston, Massachusetts, USA), was used. Statistical significance was set at *p* < 0.05.

## 3. Results

### 3.1. Recruitment and Baseline Characteristics

A total of 120 participants were allocated to the intervention (*n* = 60) and the control (*n* = 60) groups. There were no statistically significant differences in baseline characteristic between the groups. Table 1 shows the baseline characteristics of the participants.

### 3.2. Participant Flow Diagram and Outcome Measurements

**Figure 2:**
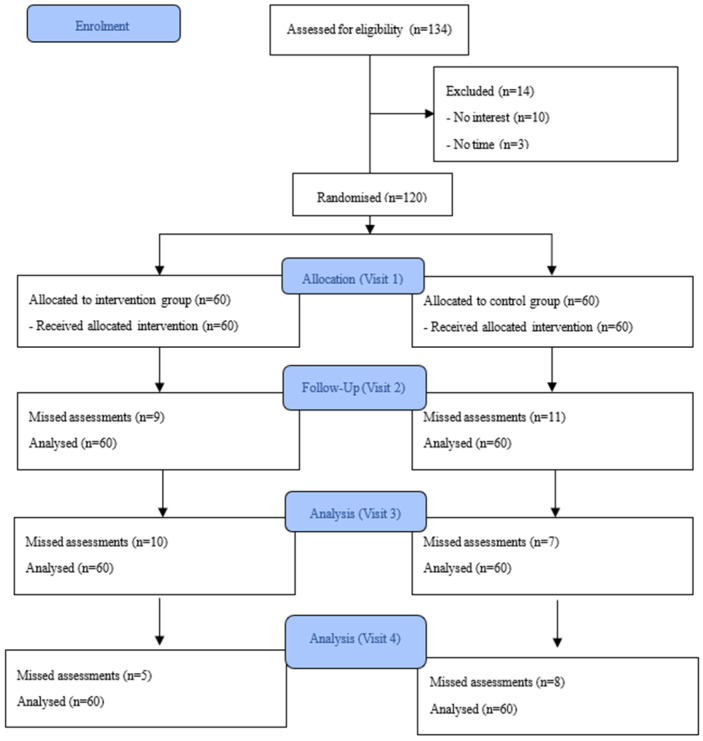
Participant flow diagram.

Figure 2 illustrates the participant flow. A total of 5–8 participants in both groups missed assessments at visits 2, 3, and 4. The final analyses were performed among 60 participants in each group per the intention-to-treat analysis. There were no adverse events reported related to study participation in both groups.

### 3.3. Primary and Secondary Outcomes

At visit 2 (follow-up measurements), the proportion of participants meeting PA recommendations was significantly different between the intervention and control groups across aerobic activity (23.3% vs. 6.7%, RR 3.5, 95%CI 1.2 to 10.0, *p* < 0.05), muscle strengthening (31.7% vs. 0, RD 0.3, 95%CI 0.2 to 0.4, *p* < 0.001), and multicomponent activity (20.0% vs. 0, RD 0.2, 95%CI 0.1 to 0.3, *p* < 0.001). The median time spent in MVPA per week was different between the intervention and control groups (90 min/week vs. 60 min/week, Cliff’s delta −0.214, 95%CI −0.402 to −0.009, *p* < 0.05). No significantly different changes in body composition measures or physical fitness tests were found between the two groups (Table 2).

At visit 3 (end-point measurements), the proportion of participants meeting PA recommendations was not significantly different between the intervention and control groups across aerobic activity (15.0% vs. 15.0%, RR 1.0, 95%CI 0.4 to 2.3, *p* = 1.000), muscle strengthening (8.3% vs. 6.7%, RR 1.2, 95%CI 0.4 to 4.4, *p* = 1.000), and multicomponent activity (3.3% vs. 5.0%, RR 0.7, 95%CI 0.1 to 3.8, *p* = 1.000). The median time spent in MVPA per week was not significantly different between the intervention and control groups (85 min/week vs. 60 min/week, Cliff’s delta −0.021, 95%CI −0.224 to 0.018, *p* = 0.844). The body compositions were not significantly different between the two groups. Percentage of participants who past flexibility test was different between the intervention and control groups (60.0% vs. 41.7%, RR 1.4, 95%CI 1.0 to 2.1, *p* < 0.05) (Table 2).

At visit 4 (continuing measurements), the proportion of participants meeting PA recommendations was not significantly different between the intervention and control groups across aerobic activity (30.0% vs. 26.7%, RR 1.1, 95%CI 0.6 to 2.0, *p* = 0.685), muscle strengthening (6.7% vs. 5.0%, RR 1.3, 95%CI 0.3 to 5.7, *p* = 1.000), and multicomponent activity (3.3% vs. 1.7%, RR 2.0, 95%CI 0.2 to 21.5, *p* = 1.000). The median time spent in MVPA per week was not significantly different between the intervention and control groups (90 min/week vs. 90 min/week, Cliff’s delta −0.075, 95%CI −0.276 to 0.131, *p* = 0.475). The body compositions and physical fitness tests were not significantly different between the two groups (Table 2).

### 3.4. Longitudinal Changes in Outcomes

Time spent in MVPA differed significantly across the four visits (*p* < 0.001) but not between the groups (Table 3). In both groups, time spent in MVPA increased significantly from visit 1 to visit 4 (intervention group: mean difference 72.4 min/week, 95%CI 27.4 to 117.4, *p* < 0.001; control group: mean difference 56.1 min/week, 95%CI 11.0 to 101.1, *p* = 0.04) (Table 4).

## 4. Discussion

Our work demonstrated that a sports scientist, co-located in a primary care setting as part of an integrated healthcare team, may have a positive impact on short-term improvements in patient PA levels. The differences between the intervention and control groups had ceased after the first follow-up. Compared to baseline, time spent in aerobic PA increased in both groups at visit 2 (follow-up), visit 3 (end-point), and visit 4 (continuing).

The PASS programme effectively facilitated participants to achieve PA goals in the first 3–4 months, while the effectiveness between the intervention and control groups were not different in a longer period. The effectiveness of PA interventions in primary care settings was different across studies [1,20]. A systematic review reported longer interventions (e.g., 6 to 12 months) were more effective than shorter interventions [20]. However, our study showed contrasting findings, in which the effectiveness of the intervention was not observed through the follow-up periods. Two possible mechanisms may explain this. First, the intensity of the intervention was reduced between visits 2 and 3; there was no tailored PA programme by a sports scientist at visit 2. At visit 2, the sports scientist only met each participant and measure primary and secondary outcomes. The sports scientist did not remind the tailored PA programme assigned at visit 1 for the intervention group to avoid potential measurement bias. Second, time spent in weekly aerobic PA and percentage of participants who met the PA recommendations increased in both groups. Participants in both groups were screened for current PA at every visit. This led to physician’s PA counselling during the consultations and likely contributed to changes in PA in both groups.

The entire process, which included PA screening by a nurse, brief PA counselling by a physician, and outcome measurement by a sports scientist, had the potential to improve aerobic PA in both groups. We designed the process as usual care, which participants in both groups received equally. Ideally, for a research setting, outcome measurements should not be performed by the sports scientist who also designed the tailored PA programme for the intervention group. However, in the real-world setting, it was more feasible for the sports scientist to measure the outcomes. Aerobic PA was prioritised and well-perceived recommendation, while muscle strengthening, balance, and multicomponent activities were often under-emphasised [21,22]. Although the PASS programme included advice on strengthening and multicomponent PA, the percentage of meeting the recommendation remained low. Most participants in the intervention group had not continued using resistance bands provided in the study. In addition, they rarely sought alternative equipment or resources. The findings of this study also reflect a challenge for PA promotion in primary care settings to impact strengthening and multicomponent PA according to the current international PA guidelines [14,23].

This study did not show significant changes in body composition. A systematic review of RCTs reported a small but statistically significant reduction in BMI (0.21 kg/m^2^) following PA promotion interventions in primary care, with a baseline BMI of 29.2 kg/m^2^ [24]. However, this change may not represent clinically meaningful weight loss. For clinical significance, a 5% to 10% reduction in body weight is generally expected [25,26]. For example, a person weighing 80 kg and standing 1.70 m tall has a BMI of 27.7 kg/m^2^. A 5% to 10% reduction in body weight would result in a BMI of approximately 26.3 kg/m^2^ to 24.9 kg/m^2^, requiring a reduction of 1.4 to 2.8 kg/m^2^. This scenario suggests that PA promotion alone in our study may not be sufficient to achieve clinically significant changes in body composition. However, a systematic review revealed the importance of achieving 150 to 300 min/week of MVPA to achieve clinically important reductions in body composition [27]. Dietary interventions are another component that can be combined with PA interventions to improve body composition [28]. Future studies that focus on improvements of body composition may need to address dietary issues alongside PA.

Regarding the type and intensity of PA, a study reported significant reductions in fat mass among participants engaged in structured exercise programmes, such as active commuting by bike, moderate-intensity leisure-time exercise, and vigorous-intensity leisure-time exercise [29]. Greater effects were observed in the vigorous-intensity group [29]. At one-year follow-up after the 6-month intervention, participants in the active commuting and vigorous-intensity exercise groups maintained improvements in body composition [30]. This highlights the importance of PA intensity in achieving meaningful changes in body composition.

This study was conducted in a hospital-based clinic in a university hospital. This setting may differ from other primary care environments. However, the clinic setting in this study is comparable to other hospital-based clinics in the public sector and to services provided in community health centres in remote areas in Thailand, in terms of NCD care characteristics. In addition, the study was conducted in a real-world routine service, rather than an idealised research setting, making it adoptable and adaptable for other primary care settings. The team-based and co-location approach designed for this study requires further consideration. First, the availability of sports scientists in primary care is a key limitation, necessitating the development of healthcare infrastructure and human resource management. Second, financial issues are another important concern for supporting PA promotion, such as incentives for healthcare insurers and providers to deliver PA promotion [31,32].

The PASS programme proposed a model for integrating the expertise of sports scientists into the primary care setting at the point of care. This approach may serve as a potential alternative or complement to traditional physical activity referral schemes (PARS). PARS have been recognised as interventions to increase PA participation [33]. They often act as connectors between healthcare professionals and allied health professionals or community-based PA advisors [34]. Various forms and components of PARS have been implemented across countries and settings [35,36,37]. In this study conducted in Thailand, PARS are not yet a common practice. The system and intervention to support PA promotion were designed based on a hospital-based clinic, without access to community-based PA specialists. Moreover, sports scientists in Thailand are not certified to provide care for clinical populations [12].

### 4.1. Clinical Implications

This study illustrates an example of embedding PA promotion into clinical practice by integrating the roles and expertise of a multidisciplinary team using a team-based approach. This serves as an alternative to exercise referral schemes, which require external resources. However, sports scientists are not commonly part of primary care teams. In real-world settings, sports scientists may be invited to work with the primary care team for a half-day per week, and not all patients will require their involvement.

The findings of this study did not demonstrate long-term differences between the intervention and control groups in relation to the involvement of a sports scientist. The entire process, including PA screening by a nurse, brief counselling by a physician, and consultation with a sports scientist, was associated with changes in patients’ PA levels. This study highlighted the long-term improvement in time spent in MVPA in both groups. These findings suggest that assessing PA participation can serve as a starting point to raise awareness of PA promotion among both patients and healthcare providers. In addition, integrating the team-based care and a co-location model into routine services is a potential approach to promote PA in clinical settings.

To improve effectiveness, intervention intensity should be tailored to individual needs rather than applying a fixed programme for all patients. Lastly, although the programme improved aerobic PA levels, promoting strengthening and multicomponent PA remains a challenge in clinical settings. There is a need to develop interventions and strategies that encourage patients to engage more in these types of activities.

### 4.2. Limitations

First, the measurement of PA participation relied on a self-reported method, which may lead to recall bias. Using tools for objective measurements, such as accelerometers, would strengthen the measurement and conclusions of the study. However, access to and management of accelerometers were not feasible for our participants and timeline of the study. A subjective measurement method using a set of brief questions was practical in our setting. Although this method is less valid than objective measurement using an accelerometer, the use of the EVS, which takes less than 30 s, is more feasible in clinical settings [38]. Second, outcome measurement was conducted by a sports scientist in both groups. This may have influenced PA motivation in the control group, despite the absence of a sports scientist-led intervention. Third, PA levels in both groups may have been affected by weather conditions. A majority of participants in both groups reported less PA participation at follow-ups during the rainy season. Fourth, this study did not compare factors associated with PA improvements between participants with significantly increased PA and those with minimal changes in PA.

## Figures and Tables

**Table 1:** Baseline characteristics of the participants.

Characteristic	Total (*n* = 120)	Intervention (*n* = 60)	Control (*n* = 60)	*p*-Value
**Age** (median, IQR) [years]	56.5 (50.0 to 62.0)	55.5 (48.0 to 63.0)	58.0 (52.0 to 61.0)	0.670 ^†^
**Sex**				0.166 ^‡^
Female (*n*, (%))	83 (69.2)	45 (75.0)	38 (63.3)	
Male (*n*, (%))	37 (30.8)	15 (25.0)	22 (36.7)	
**NCDs**				
T2DM (*n*, (%))	21 (17.5)	11 (18.3)	10 (16.7)	0.810 ^‡^
HT (*n*, (%))	41 (34.2)	24 (40.0)	17 (28.3)	0.178 ^‡^
DLP (*n*, (%))	117 (97.5)	58 (96.7)	59 (98.3)	1.000 ^‡^
**MVPA** (median, IQR) [min/week]	0 (0 to 90)	7.5 (0 to 90)	0 (0 to 75)	0.731 ^†^

DLP, dyslipidaemia; HT, hypertension; IQR, interquartile range, MVPA, moderate- to vigorous-intensity physical activity; T2DM, type 2 diabetes mellitus. ^†^ Mann–Whitney U test. ^‡^ Chi-square test or Fisher’s exact test.

**Table 2:** Outcome measurements using an intention-to-treat analysis (visits 1 to 4).

Outcome	Visit 1: Baseline (Month 0)			Visit 2: Follow-Up (Months 3–4)			Visit 3: End-Point (Months 6–8)			Visit 4: Continuing (Months 9–12)		
	Intervention	Control	*p*-Value	Intervention	Control	*p*-Value	Intervention	Control	*p*-Value	Intervention	Control	*p*-Value
Loss to follow-up (*n*, (%))	Allocation			9 (15.0)	11 (18.3)		10 (16.7)	7 (11.7)		5 (8.3)	8 (13.3)	
Number of participants for analysis	60	60		60	60		60	60		60	60	
**Primary outcomes**												
Meeting PA recommendations												
Aerobic (*n*, (%))	0 (0)	0 (0)	1.000 ^‡^	14 (23.3)	4 (6.7)	**0.011 ^‡^**	9 (15.0)	9 (15.0)	1.000 ^‡^	18 (30.0)	16 (26.7)	0.685 ^‡^
Muscle-strengthening (*n*, (%))	0 (0)	0 (0)	1.000 ^‡^	19 (31.7)	0	**<0.001 ^‡^**	5 (8.3)	4 (6.7)	1.000 ^‡^	4 (6.7)	3 (5.0)	1.000 ^‡^
Multicomponent (*n*, (%))	0 (0)	1 (1.7)	1.000 ^‡^	12 (20.0)	0	**<0.001 ^‡^**	2 (3.3)	3 (5.0)	1.000 ^‡^	2 (3.3)	1 (1.7)	1.000 ^‡^
MVPA (median, IQR) [min/week]	7.5 (0, 90)	0 (0, 75)	0.731 ^†^	90.0 (30.0, 120.0)	60.0 (0, 92.5)	**0.040 ^†^**	85.0 (0, 120.0)	60.0 (27.5, 120.0)	0.844 ^†^	90.0 (43.8, 150.0)	90.0 (37.5, 142.5)	0.475 ^†^
**Secondary outcomes**												
***Body composition***												
Body weight (median, IQR) [kg]	60.4 (52.0, 67.2)	64.1 (56.9, 70.9)	0.097 ^†^	61.6 (52.0, 67.4)	63.8 (56.6, 71.8)	0.127 ^†^	61.3 (52.1, 68.4)	62.4 (56.5, 71.5)	0.159 ^†^	60.9 (53.0, 67.7)	63.8 (57.7, 71.8)	0.105 ^†^
Body mass index (median, IQR) [kg/m^2^]	24.2 (22.2, 26.9)	24.6 (22.8, 27.0)	0.342 ^†^	24.2 (21.7, 27.1)	24.5 (23.0, 27.1)	0.388 ^†^	24.5 (22.0, 27.2)	24.5 (22.8, 27.0)	0.492 ^†^	24.4 (22.0, 27.0)	24.6 (23.2, 26.6)	0.446 ^†^
Fat mass (median, IQR) [kg]	18.4 (13.4, 25.2)	19.1 (15.4, 22.9)	0.650 ^†^	17.7 (13.8, 25.1)	19.4 (15.4, 22.9)	0.635 ^†^	18.2 (13.5, 25.4)	19.4 (15.3, 23.0)	0.616 ^†^	19.2 (13.9, 25.4)	19.9 (15.7, 23.8)	0.650 ^†^
Percentage of fat (median, IQR) [%]	31.0 (24.6, 37.6)	31.2 (24.1, 36.4)	0.605 ^†^	31.0 (25.6, 36.8)	31.0 (24.6, 36.3)	0.759 ^†^	31.7 (24.4, 36.0)	30.7 (24.0, 36.1)	0.625 ^†^	32.4 (25.2, 37.6)	31.6 (25.0, 36.5)	0.601 ^†^
Muscle mass (median, IQR) [kg]	37.4 (34.6, 42.6)	39.7 (35.7, 48.0)	0.071 ^†^	37.4 (34.6, 42.6)	39.2 (35.0, 47.8)	0.143 ^†^	38.2 (34.6, 43.0)	39.2 (35.2, 48.4)	0.131 ^†^	37.8 (34.8, 42.6)	39.6 (36.1, 47.0)	0.055 ^†^
Percentage of muscle mass (median, IQR) [%]	64.8 (58.7, 71.5)	65.1 (59.9, 71.8)	0.596 ^†^	65.1 (59.7, 70.8)	65.0 (60.0, 71.6)	0.755 ^†^	64.6 (60.2, 71.4)	64.9 (60.0, 71.8)	0.864^†^	63.9 (58.8, 70.9)	64.3 (59.4, 71.1)	0.696 ^†^
***Physical fitness tests (passing scores)***												
Flexibility (*n*, (%))	17 (28.3)	11 (18.3)	0.195 ^‡^	32 (53.3)	24 (40.0)	0.143 ^‡^	36 (60.0)	25 (41.7)	**0.045 ^‡^**	40 (66.7)	31 (51.7)	0.095 ^‡^
Muscle strength and endurance (*n*, (%)	32 (53.3)	27 (45.0)	0.361 ^‡^	36 (60.0)	36 (60.0)	1.000 ^‡^	29 (48.3)	29 (48.3)	1.000 ^‡^	38 (63.3)	33 (55.0)	0.353 ^‡^
Cardiovascular endurance (*n*, (%))	54 (90.0)	44 (73.3)	**0.018** ^‡^	55 (91.7)	53 (88.3)	0.543 ^‡^	42 (70.0)	42 (70.0)	1.000 ^‡^	44 (73.3)	38 (63.3)	0.239 ^‡^

IQR, interquartile range, MVPA, moderate- to vigorous-intensity physical activity. ^†^ Mann–Whitney U test. ^‡^ Chi-square test or Fisher’s exact test. Flexibility tests: sit and reach for individuals 35-59 years of age; back scratch for individuals 60–70 years of age. Muscle strength and endurance tests: 60 s chair stand for individuals 35–59 years of age; 30 s chair stand for individuals 60–70 years of age. Cardiovascular endurance test: 3 min step up and down for individuals 35–59 years of age; 2 min step up and down for individuals 60-70 years of age. Bold numbers present statistical significance.

**Table 3:** Analysis of variance of moderate- to vigorous-intensity physical activity.

Source of Variation	Degrees of Freedom	Sum Square	Mean Square	F	*p*-Value
Visit	3	260,754	86,918	13.246	**<0.001**
Group	1	11,653	11,653	1.776	0.183
Visit * Group	3	9471	3157	0.481	0.696
Error	472	3,097,195	6562		

Bold numbers present statistical significance.

**Table 4:** Post hoc comparisons of moderate- to vigorous-intensity physical activity.

Group and Visit	Mean Difference	95% Confidence Interval		*p*-Value
		Lower Bound	Upper Bound	
**Intervention group**				
visit 2–visit 1	49.2	4.1	94.2	**0.021**
visit 3–visit 1	43.1	−1.9	88.1	0.072
visit 4–visit 1	72.4	27.4	117.4	**<0.001**
visit 3–visit 2	−6.1	−51.1	38.9	1.000
visit 4–visit 2	23.2	−21.8	68.3	0.767
visit 4–visit 3	29.3	−15.7	74.4	0.494
**Control group**				
visit 2–visit 1	31.9	−13.1	76.9	0.379
visit 3–visit 1	44.9	−0.1	89.9	0.051
visit 4–visit 1	56.1	11.0	101.1	**0.004**
visit 3–visit 2	13.0	−32.0	58.0	0.988
visit 4–visit 2	24.2	−20.9	69.2	0.729
visit 4–visit 3	11.2	−33.9	56.2	0.995

Bold numbers present statistical significance.

## Data Availability

The original contributions presented in this study are included in the article. Further inquiries can be directed to the corresponding author.
